# Positive Chemotaxis of the Entomopathogenic Nematode *Steinernema australe* (Panagrolaimorpha: Steinenematidae) towards High-Bush Blueberry (*Vaccinium corymbosum*) Root Volatiles

**DOI:** 10.3390/ijms241310536

**Published:** 2023-06-23

**Authors:** Ricardo Ceballos, Rubén Palma-Millanao, Patricia D. Navarro, Julio Urzúa, Juan Alveal

**Affiliations:** 1 Laboratory of Insects Chemical Ecology, Instituto de Investigaciones Agropecuarias, INIA Quilamapu, Av. Vicente Méndez 515, Chillán 3800062, Chile; 2 Laboratory of Insects Science, Instituto de Investigaciones Agropecuarias, INIA Carillanca, Km 10, Camino Cajón-Vilcún, Temuco 4800000, Chile; patricia.navarro@inia.cl; 3 Vicerrectoría de Investigación y Postgrado, Universidad de La Frontera, Temuco 4811230, Chile

**Keywords:** root volatiles, belowground interactions, entomopathogenic nematode recruitment, foraging behavior, olfactometer

## Abstract

The foraging behavior of the infective juveniles (IJs) of entomopathogenic nematodes (EPNs) relies on host-derived compounds, but in a tri-trophic context, herbivore-induced root volatiles act as signals enhancing the biological control of insect pests by recruiting EPNs. In southern Chile, the EPN *Steinernema australe* exhibits the potential to control the raspberry weevil, *Aegorhinus superciliosus,* a key pest of blueberry *Vaccinium corymbosum*. However, there is no information on the quality of the blueberry root volatile plume or the *S. australe* response to these chemicals as putative attractants. Here, we describe the root volatile profile of blueberries and the chemotaxis behavior of *S. australe* towards the volatiles identified from *Vaccinium corymbosum* roots, infested or uninfested with *A. superciliosus* larvae. Among others, we found linalool, α-terpineol, limonene, eucalyptol, 2-carene, 1-nonine, 10-undecyn-1-ol, and methyl salicylate in root volatiles and, depending on the level of the emissions, they were selected for bioassays. In the dose–response tests, *S. australe* was attracted to all five tested concentrations of methyl salicylate, 1-nonine, α-terpineol, and 2-carene, as well as to 100 µg mL^−1^ of 10-undecyn-1-ol, 0.1 and 100 µg mL^−1^ of linalool, and 100 µg mL^−1^ of limonene, whereas eucalyptol elicited no attraction or repellency. These results suggest that some volatiles released from damaged roots attract *S. australe* and may have implications for the biocontrol of subterranean pests.

## 1. Introduction

Plants constitutively produce volatile organic compounds (VOCs), which are released by leaves, fruits, and roots and whose profile may change in response to abiotic conditions (i.e., temperature, nutrients, and radiation) and biotic factors (herbivory and pathogen attack) [1]. VOCs, triggered by herbivory and pathogens, often include the so-called herbivore-induced plant volatiles (HIPVs), with HIPVs emitted by the aerial parts of the plant receiving much more attention than those released by belowground tissues. HIPVs act as induced defenses, attracting natural enemies of herbivores, which mainly belong to terpenoids, jasmonates, and aromatic compounds [2]. Moreover, some HIPVs are only biosynthesized in response to herbivory, such as sesquiterpene (*E*)-β-caryophyllene [3], α-santalene, and α-*Z*-bergamotene [4], or could be expressed as the overproduction of constitutive volatiles [5]. HIPVs emitted by belowground tissues are still poorly documented in the scientific literature [6], even though several major crop pests uniquely feed on roots [7]. Root HIPVs have been addressed in maize *Zea mays* L. (Poaceae) [3], milkweed *Asclepias syriaca* L. (Apocynaceae) [5], citrus grapefruit *Citrus paradisi* Macf. (Rutaceae), *Ruta graveolens* L. (Rutaceae) [8], apple *Malus domestica* Borkh. (Rosaceae) [9], spotted knapweed *Centaurea stoebe* L. (Asteraceae) [10], cotton *Gossypium herbaceum* (Malvaceae) [11], and tomato *Solanum lycopersicum* L. (Solanaceae) [12], among others. Given that HIPVs are released as a mixture of compounds, it is important to highlight that some of them could be produced and released by the plant due to herbivory, but others could also be repressed for the same reason [13].

Natural enemies such as predators, parasitoids, and pathogens, including entomopathogenic nematodes (EPNs), exploit HIPVs in tri-trophic interactions [9,14,15,16] inducing their chemotaxis to chemical-based stimuli [17]. Rivera et al. [18] studied the potential of adding (*E*)-β-caryophyllene and pregeijerene to the soil matrix to enhance its attraction to EPNs. In this study, the efficacy of *Steinernema glaseri* Steiner (Rhabditida: Steinermatidae) was evaluated against the oriental beetle *Anomala orientalis* Waterhouse (Coleoptera: Scarabaeidae) in a commercial blueberry orchard. The results under laboratory conditions showed that compound (*E*)-β-caryophyllene was significantly more attractive than pregeijerene to EPNs; however, under field conditions, the differences were less meaningful. In another study, Degenhardt et al. [19] reported the ability to produce (*E*)-β-caryophyllene in a non-emitting maize line (transformed with an (*E*)-β-caryophyllene-synthase gene), resulting in significantly less damage to roots by *Diabrotica virgifera virgifera* LeConte (Coleoptera: Chrysomelidae).

Research on blueberry VOCs has been mainly focused on the characterization of the fruit aroma [20] and other aboveground structures, such as the flowers and leaves [21,22]. In Chile, the high-bush blueberry *Vaccinium corymbosum* (Ericaceae) is an economically important crop, with more than 100,000 tons exported annually. In the country, this crop faces economically important pest problems mainly caused by the native weevil *Aegorhinus superciliosus* (Coleoptera: Curculionidae). The larvae of *A*. *superciliosus* feed on roots, tunneling galleries, and causing irreversible damage, which leads to plant death and even the death of the entire orchard [23]. There has been no effective control against the larva because of the gallery’s cryptic location inside the root. So far, using EPNs has been proposed as a promising management tool due to their ability to search for and find the host [24]. In recent years, the native EPN species *Steinernema australe* (Panagrolaimorpha: Steinenematidae) was evaluated against larvae of *A*. *superciliosus* with 100% and 72% efficacy under laboratory and field conditions, respectively [25]. Later, Navarro et al. [26] evaluated the same isolate of *S*. *australe* against *Aegorhinus nodipennis* (Coleoptera: Curculionidae) in blueberry and sarsaparilla orchards showing 68% and 65% mortality, respectively. Edgington et al. [27] reported *Steinernema australe* as a new species of Steinernematidae isolated from a soil sample collected from Magdalena Island in the Chilean Patagonian region. This study provided baseline data on the biology and ecology of this species, highlighting its relatively rapid lifecycle and ability to infest a host at relatively low temperatures [28], two aspects of great interest considering its potential to be used in pest management. *S*. *australe* is well-adapted to the territory where *A*. *superciliosus* is present; however, little is known regarding plant–insect–EPN interactions considering the root blueberry model of study. In this study, we hypothesized that *S*. *australe* would be attracted to the volatiles released by damaged blueberry roots when *A. superciliosus* larvae attack them. To test this hypothesis, we (1) identified the volatiles emitted from roots of *V. corymbosum* uninfested and infested with *A. superciliosus* larvae and (2) determined the chemotaxis of the *S. australe* infective juveniles (IJs) in response to the volatiles emitted by uninfested and damaged roots.

## 2. Results

### 2.1. Root Volatiles

We successfully collected the volatiles from *A*. *supercilious*-damaged blueberry roots by Headspace combined with SPME. Along with the identification achieved by the GC-MS, we estimated the relative abundance of the compounds in the volatile profile by comparing between larvae-infested and uninfested roots (Table 1). We determined eighteen VOCs from the uninfested and infested samples of *V. corymbosum* roots.

### 2.2. Nematode Response to Root Volatiles: Chemotaxis Assay

**Figure 1:**
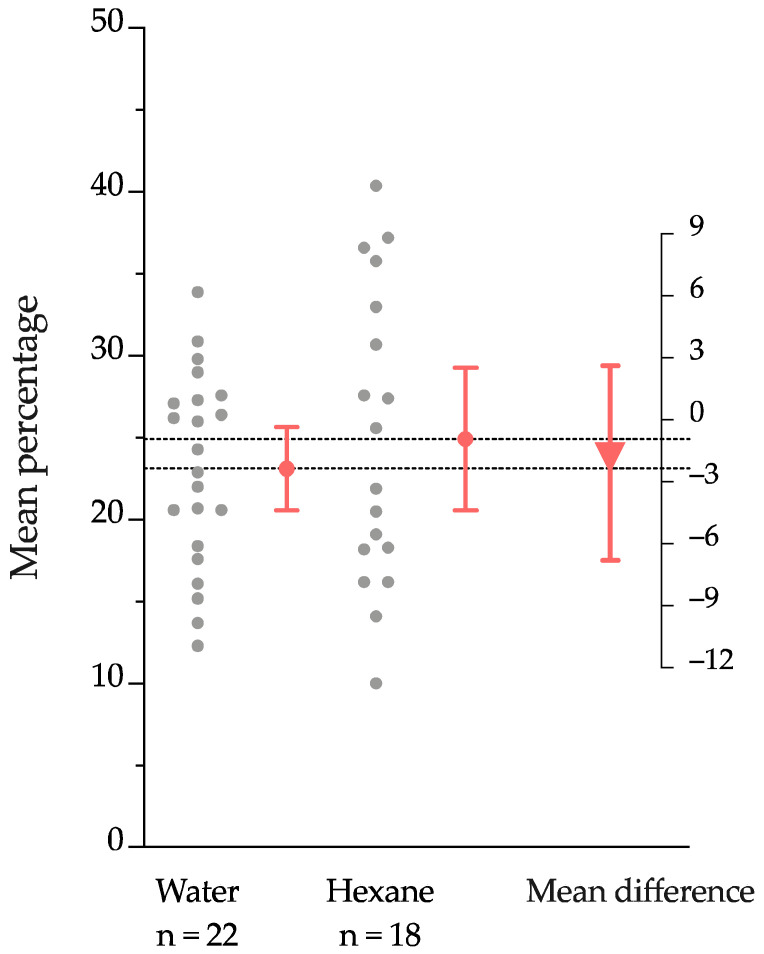
The Gardner–Altman estimation plot for the percentage of IJs collected in the water and the hexane. The grey dots represent the replicates, and the vertical red solid lines and the red dots represent the confidence intervals at 95% and their respective means, respectively. The effect size for the mean difference is shown (right scale), and the triangle and its vertical line represent the estimated difference between the water and the hexane. The bootstrapping confidence interval (95%) was obtained by a two-sided permutation *t*-test with 5000 permutations.

We investigated the impact of the selected VOCs (Table 1) released by the uninfested and infested blueberry roots on the chemotaxis of the *S. australe* IJs using authentic commercial standards in a two-choice bioassay. We expressed the attraction of the IJs to the selected VOCs as the mean difference between the percentage of IJs in the treatment and control areas. Thereby, we showed the positive values as the attraction of IJs for a dose of treatment over the control, and if its corresponding 95% confidence interval did not include the value 0%, the difference was considered significant (*p* < 0.05). Hexane, which was used to dilute the commercial standards, was previously evaluated to confirm no substantial differences in the dispersal and survival of IJs. Both the hexane and water solution were compared to discard any bias in the chemotaxis assay (Figure 1).

**Figure 2:**
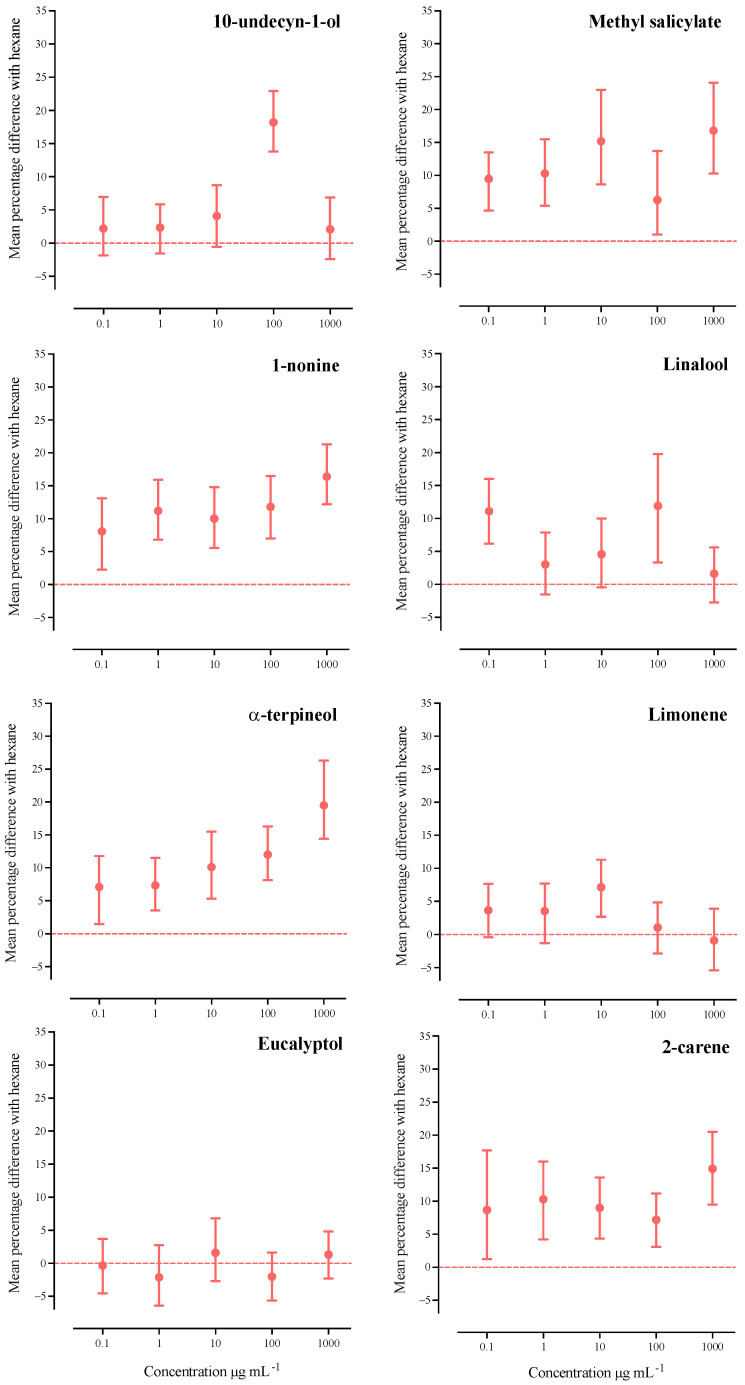
Dose–response chemotaxis trials. The mean difference (%) of the IJs collected from the olfactometric bioassays between the compound dose and control (±95% CI). The positive values indicate the IJs’ attraction to the dose of the corresponding treatment, and when it is a 95% confidence interval and does not include the value 0%, the difference is considered significant (*p* < 0.05). Zero represents the control (horizontal dotted line). We estimated the confidence intervals by a two-sided permutation *t*-test with 5000 permutations.

In the dose–response chemotaxis assays (Figure 2), we observed that the IJs of *S. australe* were significantly attracted to 100 µg mL^−1^ of 10-undecyn-1-ol, resulting in 18.2% more IJs in the treatment area than for the control (95% CI: 13.8–22.9%; *t*_45_ = 7.1180; *p* = 0.0001). All other tested doses of 10-undecyn-1-ol were not significantly different from the control. All five tested concentrations of methyl salicylate elicited significant attraction from the IJs of *S. australe*, ranging from 9.48% more IJs collected at 0,1 µg mL^−1^ (95.0% CI: 4.67–13.5%; *t*_31_ = 4.1657; *p* = 0.0002) up to 16.8% at 1000 µg mL^−1^ (95.0% CI: 10.3–24.1%; *t*_31_ = 4.9710; *p* = 0.0002) when compared to the control. However, at 100 µg mL^−1^, the attractant effect dropped to 6.28% (95.0% CI: 1.02–13.7%; *t*_31_ = 2.0301; *p* = 0.0484), although still positive and significantly higher compared to the control. Similarly, 1-nonine elicited a positive dose–response effect on the chemotaxis of IJs, ranging from 8.07% more IJs (95.0% CI: 2.25–13.1%; *t*_33_ = 2.8358; *p* = 0.0072) at 0.1 µg mL^−1^ to 16.4% (95.0% CI: 12.2–21.3%; *t*_31_ = 6.9081; *p* = 0.00001) at 1000 µg mL^−1^. When *S*. *australe* IJs confronted linalool, we only found a significant attraction at 0.1 and 100 µg mL^−1^, with 11.1% (95.0% CI: 6.16–16.0%; *t*_31_ = 4.3573; *p* = 0.00001) and 11.9% (95.0% CI: 3.33–19.8%; *t*_31_ = 2.9201; *p* = 0.0006) more IJs, respectively. The percentage of attraction elicited by α-terpineol on *S. australe* rose as we increased the dose, from 7.08% more IJs (95.0% CI: 1.49–11.8%; *t*_31_ = 2.6846; *p* = 0.0128) at 0.1 µg mL^−1^ to 19.5% more IJs at 1000 µg mL^−1^ (95.0% CI: 14.4–26.3%; *t*_31_ = 6.5385; *p* = 0.00001). Unlike 10-undecyn-1-ol, methyl salicylate, and 1-nonine, the α-terpineol, limonene, and eucalyptol barely elicited chemotaxis to *S*. *australe* IJs. We found that limonene only at 10 µg mL^−1^ induced a significant attraction to *S*. *australe,* with an increase of 7.13% more IJs (95.0% CI: 2.68–11.3%; *t*_31_ = 3.1022; *p* = 0.0058). All doses of 2-carene evoked positive chemotaxis on *S. australe*. The higher chemotaxis observed with this compound showed 15% more IJs collected from the treatment area (95.0% CI: 9.5–20.5%; *t*_31_ = 5.2690; *p* = 0.00001) at 1000 µg mL^−1^ than the control area.

## 3. Discussion

To the best of our knowledge, the volatiles released by the roots of *V*. *corymbosum* have not yet been explored. In this study, we demonstrated that *V. corymbosum* root volatiles comprise a complex blend of VOCs altered by the herbivory of *A*. *supercilious* larva. Through this work, we contributed to increasing the list of compounds reported as volatiles emitted by *V. corymbosum*, either constitutive or induced by herbivory. VOCs from *V*. *corymbosum* have been extensively studied in aboveground systems, including the VOCs released by flowers, fruits, and leaves [20,29,30,31,32,33,34]. It should also be noted that, typically, the volatile root profile triggered by herbivory may differ from the aerial profile of the plant [35]. In addition, we demonstrated that some of the root volatiles evaluated in this study elicited chemotaxis to *S. australe* IJs, a promising entomopathogen for the key pest *A*. *superciliosus* in the blueberry crop in the south of Chile.

Our results showed that the blend of VOCs released by the *V. corymbosum* roots comprised terpenoids, esters, aliphatic hydrocarbons, alcohols, ketones, and other compounds. Terpenes compose the most chemically and structurally diverse family of natural products [36]. Their release by damaged roots has been shown to recruit natural enemies of herbivores [37]. This belowground tri-trophic interaction was described in the seminal work of Rasmann et al. [3], where maize roots released the sesquiterpene (*E*)-β-caryophyllene in response to feeding of *D. virgifera virgifera*. In this respect, our results showed a large increase in the emission of 2-carene, α-terpineol, linalool, and limonene and a moderate increase in the emission of cis-myrtanol and 3-octanone. We also observed a diminution of 4,8-dimethyl-1,7-nonadien-4-ol, eucalyptol, and myrcenol. Abraham et al. [9] found that linalool was reduced in *Malus domestica* roots in response to *Melolontha melolontha* L. (Coleoptera: Scarabaeidae) larval feeding. In the same study, these authors reported the emission of methyl salicylate in response to root herbivory being absent in the control plants. In contrast, we found a large reduction in the emission of methyl salicylate in response to root herbivory by *A. superciliosus* in *V. corymbosum*. The direct response of EPNs toward methyl salicylate has not been intensely studied in the scientific literature. In citrus plants, foliar application of this compound acted as an elicitor of HIPVs in roots, resulting in recruitment of the EPN *Steinernema diaprepesi* Nguyen & Duncan (Rhabditida: Steinernematidae) [38,39]. In our bioassays, the attraction of *S. australe* to all five tested concentrations of methyl salicylate suggests that the IJs positively respond to this compound, even at low concentrations.

On the other hand, 10-undecyn-1-ol increased in *V. corymbosum* roots in response to *A. superciliosus* herbivory. The presence of this alcohol in plant extracts has been associated either with antimicrobial activity [40] or as a precursor for laboratory synthesis of moth pheromones [41,42]. However, there are no reports regarding its potential role as an EPN recruiter by plants. Our findings demonstrated the attractiveness of *S*. *australe* IJs to 10-undecyn-1-ol in chemotaxis assays, making this result an interesting subject for further studies.

In terms of the terpenoids, linalool was not abundant in either *A*. *superciliosus*-infested or -uninfested root conditions. Although linalool has been reported to have limited diffusion into the soil and to be highly susceptible to degradation [43], we decided to include linalool in our profile of selected compounds because it has been reported as an HIPV-causing mixed response upon EPNs in similar studies. For instance, in olfactometric bioassays using sand as a substrate, linalool was not attractive to *Steinernema diaprepesi*, *Steinernema riobrave* (Cabanillas, Poinar, and Raulston), or *Heterorhabditis indica* Poinar, Karunakar & David [44]. Laznik and Trdan [45] also studied the olfactometric response of EPNs toward linalool, observing that only one strain of *Steinernema carpocapsae* (Weiser) was attracted to this compound, while strains of *Steinernema feltiae* (Filipjev), *Steinernema kraussei* (Steiner), and *Heterorhabditis bacteriophora* (Poinar) were not attracted. Our study showed that *S. australe* was significantly attracted to two out of the five concentrations of linalool assayed.

The results for 1-nonyne also showed an attractive effect on *S. australe* at all tested concentrations. This compound has been poorly studied in terms of its potential role as a semiochemical compound, and there are no records focusing on its ability to elicit a response upon EPNs. However, its presence has been reported among the constituents of leaf volatiles of *Ficus vogelii* Miq. (Rosales: Moraceae) [46], as well as volatile constituents from *Brassica oleracea* var. *capitata* L. (Brassicales: Brassicaceae) extracts. The latter was studied as an attractant to the caterpillar *Pieris rapae* (L.) (Lepidoptera: Pieridae) [47] and as a precursor for the synthesis of a chemical cue found in the caterpillar *Spodoptera litura* (Fab.) (Lepidoptera: Noctuidae) to elicit prey-locating behavior in the predatory stink bug *Eocanthecona furcellata* (Wolff) (Hemiptera: Pentatomidae) [48]. The terpenoid α-terpineol is a frequent secondary metabolite produced by plants [49] with antimicrobial activity [50]. Moreover, several studies have determined its presence in species from the genus *Vaccinium*. For example, Eichholz et al. [51] noted that α-terpineol was synthesized by *V. corymbosum* in response to the stress produced by UV-B radiation, as well as the major constituent of volatiles produced by stems of *Vaccinum arctostaphylos* L. [52]. This compound was reported as a component of the stink bug *Podisus maculiventris* Say (Hemiptera: Pentatomidae) aggregation pheromone [53] and as a component of the mountain pine beetle *Dendroctonus ponderosae* Hopkins (Coleoptera: Curculionidae) frass [54]. No records were found about the potential role of α-terpineol on EPNs’ behavior; however, the potential role of this compound on the plant parasitic nematode *Meloidogyne incognita* (Kofoid and White) (Tylenchida: Heteroderidae) galling was reported in cotton roots [55].

The indirect root defenses of *Asclepias syriaca* L. (Gentiananles: Apocynaceae) under the attack of the cerambycid *Tetraopes tetrophthalmus* Forster (Coleoptera: Cerambycidae) larvae showed a significantly larger release of limonene and eucalyptol than the undamaged roots, resulting in more *H. bacteriophora* attracted and less larval survival [5]. Eucalyptol was also linked to the defensive response in roots of oak trees *Quercus petraea x Quercus robur* (Fagales: Fagaceae), which was attractive to the larvae of the cockchafer *Melolontha hippocastani* Fab. (Coleoptera: Scarabaeidae) [56]. Eucalyptol produced by wounded roots of poplars *Populus trichocarpa* Torr. & A. Gray ex Hook and *P. nigra* L. (Malpighiales: Salicaceae) inhibited the growth of *Phytophtora cactorum* (Lebert & Cohn) J. Schröt. (Peronosporales: Peronosporaceae) [57] showing insecticide effects against the caterpillar *Spodoptera littoralis* (Boisduval) (Noctuidae) [58]. Despite its numerous documented effects, our results did not reveal any evidence of the behavioral role of eucalyptol on *S. australe*. Moreover, among the eight tested compounds, eucalyptol was the only compound that did not elicit a response at the assayed concentrations. For limonene, the results were slightly different. The concentration of 10 µg mL^−1^ attracted significantly more IJs than the control. Limonene is a well-known herbivory-induced volatile [52], whose presence was reported to induce attraction in adults of *A. superciliosus* [32] and to recruit *S. diaprepesi* IJs in two-choice olfactometric trials [38].

The results of the GC-MS showed increasing amounts of the monoterpene 2-carene in herbivory-attacked roots. No other studies have reported the presence of this compound in blueberry crops. However, Farag and Paré [59] observed continuous damage to tomato leaves produced by *Manduca sexta* (L.) (Lepidoptera: Sphingidae), where the emission of 2-carene was increasing. This compound was found as the most important monoterpenoid emitted in response to the attack of the leaf miner *Tuta absoluta* (Meyrick) (Lepidoptera: Gelechiidae) [60], and its presence was negatively correlated with pollinator visits in tomato flowers [61]. Our results demonstrated that 2-carene consistently elicited positive chemotaxis on the IJs of *S. australe*, suggesting that this compound may play a role as an attractant of EPNs in blueberry. Nevertheless, further studies are required to confirm this hypothesis.

The novel information obtained in this study involved *A*. *superciliosus* larva-infested root and *S*. *australe* chemical communication on blueberry. These results may contribute to a better understanding of this EPN foraging behavior in the field, considering odors not only triggered by the target pest but also by the damaged roots. Some evidence suggests that the horizontal foraging strategies of EPNs are driven by the host-finding behavior used by a given EPN species [62]. In contrast, the results obtained by Laznik and Trdan [45] in olfactometric assays suggested that the response to different volatile cues is more a strain-specific characteristic than a host-searching strategy. Interestingly, new evidence showed that the response of EPNs is not only constitutive but also the result of their previous experience [63], which may affect their host-seeking and orientation behavior [64,65].

This work aimed to contribute to the identification of volatile compounds emitted by high-bush blueberry roots infested by herbivores such as *A. superciliosus*, which were attractive to the EPN *S. australe*. Given that HIPV emissions benefit plants by recruiting pests’ natural enemies [2], the identification and manipulation of a root signal could enhance biocontrol tools for pest control belowground [8]. Moreover, further studies reported by Navarro et al. [26] supported our findings and confirmed that selected *S*. *australe* IJs improved their efficacy by 20% against *Aegorhinus nodipennis* (Coleoptera: Curculionidae) in blueberry and sarsaparilla orchards after a series of selection rounds using 2-carene as an odor stimulus. The VOC findings obtained in this work could be of interest to researchers focusing on biocontrol strategies using EPNs. However, additional studies are recommended to confirm these results under different conditions.

## 4. Materials and Methods

### 4.1. Plants and Headspace Collection of Volatiles

Eighteen-month-old *V. corymbosum* cv. Legacy plants, 50–70 cm in height, were obtained from a commercial nursery in La Union County (40°17′43″ S 73°04′56″ O) in the south of Chile. The plants were contained in 2.26 L plastic pots (12 cm diameter and 20 cm deep) with a mix of 2:1:2 peat, perlite, and oil palm fiber, and 5.5 pH. Plants were maintained under greenhouse conditions and suitably watered every two days for three months before the bioassay.

**Figure 3:**
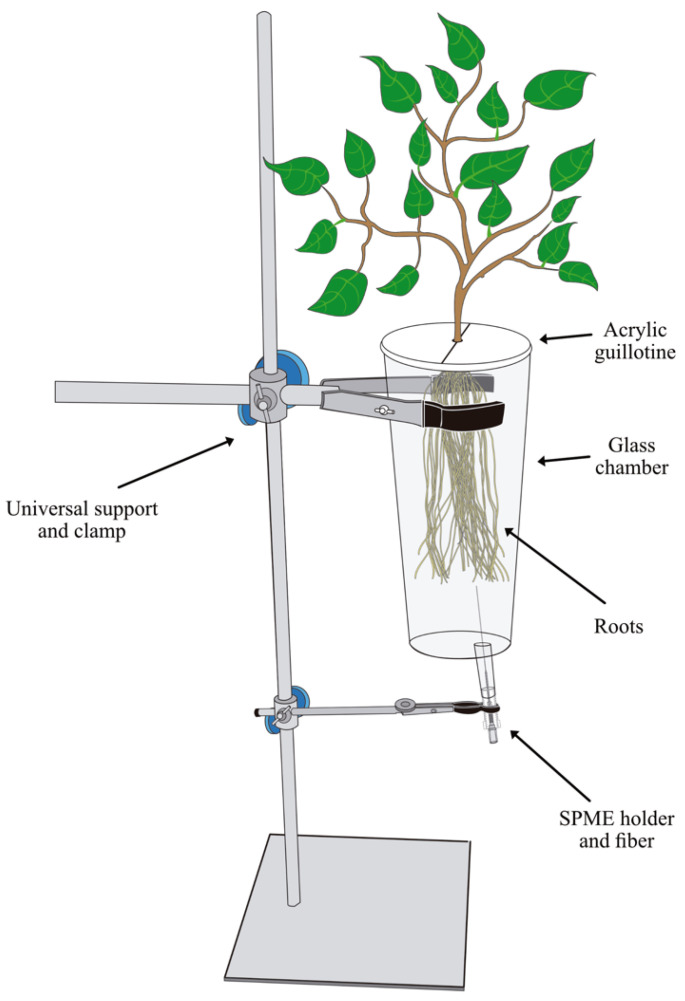
Schematic representation of the root volatile collection by solid-phase microextraction.

The headspace technique was used to collect root VOCs from uprooting *V. corymbosum* plants, as described in Rasmann et al. [5], with the following modifications. The root was gently cleaned and washed under tap water. Instead of isolating the root portion of the plant through a hole in a lab bench, we individually attached the plant to a universal stand using tweezers to enclose the roots in a 900 mL glass chamber (9.0 cm i.d. × 14.5 cm height). One end of the glass chamber was fully open to introduce the roots. Once the roots were enclosed, the aerial part was separated into a two-piece Plexiglas guillotine, which, once assembled, allowed a circular central opening for the aerial part and closure of the chamber. The other end had a small, elongated opening (0.8 cm i.d. × 2.0 cm long), where the solid-phase microextraction (SPME) fiber was inserted to sample the root volatiles (Figure 3). A manual SPME holder with polydimethylsiloxane/divinylbenzene (PDMS/DVB; df 65 μm, needle size 24 ga, Supelco) fiber was exposed to the root headspace for 30 min at 20 ± 2 °C [66,67,68]. We sampled an empty chamber as a control under the same described conditions. We randomly assigned thirty-six plants to two groups of eighteen plants each. Each plant in one group was infested with five third-instar larvae of *A. superciliosus*. The larvae fed from the root for five days, and later, the larvae and substrate were carefully removed, as described above. The other group of plants remained uninfested for the same time, and their roots were cleaned using tap water. All root volatile collections were performed without substrate or larvae. In addition, using the same technique (HS-SPME), we collected the volatiles emitted only by the *A. superciliosus* larvae, and we found none of these larval compounds in the *V. corymbosum* root volatiles.

### 4.2. Identification of the Volatile Organic Compounds (VOCs) Emitted by Roots

The SPME fibers were desorbed directly in a gas chromatographer coupled to a mass spectrometer (GC-MS QP2010 Plus; Shimadzu, Tokyo, Japan). The GC was equipped with a Restek capillary column (Rxi-5 ms: 5% dephenyl-95% dimethyl polysiloxane; 30 m × 0.25 mm ID × 1.0 μm; Restek Corp., Bellefonte, PA, USA), and the injection port was set to 250 °C. Thermal desorption was carried out in splitless mode using helium as the carrier gas at 1.0 mL min^−1^, and the oven was set at 40 °C for 1 min. Then, the temperature was increased at a rate of 5 °C min^−1^ until reaching 280 °C. The acquisition was performed in a mass range from 35 to 500 *m*/*z*, whereas ionization was performed by electron impact at 70 eV with an ion source at 200 °C [16]. The data were processed using LabSolution software (Shimadzu Corporation, Tokyo, Japan).

The identity of the collected compounds was verified by comparing their mass spectra with those in the NIST database v2.0 (National Institute of Standards and Technology, Gaithersburg, MD, USA) and with commercial standards. Compounds that had at least 90% similarity with those in the NIST database were chosen for further analysis. All chromatographic peaks in the control empty chambers were considered artifacts of the technique and discarded.

### 4.3. Culture of Entomopathogenic Nematodes (EPNs)

*Steinernema australe* IJs were obtained from a stock curated by the Microbiological Resources Bank at the Instituto de Investigaciones Agropecuarias (INIA), Quilamapu station, Chillán, Chile. The EPNs were reared in vivo, infecting the fifth instar larvae of the wax moth *Galleria mellonella* L. (Lepidoptera: Pyralidae) at the Insect Science Laboratory (INIA), Carillanca station, Vilcún, Chile. Briefly, larvae were infected with *S. australe* IJs and placed in 90 mm diameter Petri dishes containing sterile filter paper at the bottom [69]. Infected larvae were maintained in the dark at 25 °C for 72 h and transferred to modified White traps, as described by Stock and Goodrich-Blair [70]. The latter was maintained at 25 °C with 12:12 D:L, and the IJs were harvested 15 days after infection. The collected IJs were suspended in 25 mL of sterilized water and stored in 40 mL culture flasks (Thermo Scientific Nunc EasyFlask) and kept at 4 °C until use. All assays were conducted with IJs no older than seven days after harvest.

### 4.4. Odor Sources

Selected VOC commercial standards (Sigma-Aldrich Chemical Co., St. Louis, MO, USA) were used to prepare the treatment solutions at 1000, 100, 10, 1, and 0.1 µg mL^−1^. Double-distilled chromatographic grade hexane was used as the solvent. The chemicals were chosen according to the effect size computed from the GC-MS identification. Double-distilled chromatographic grade hexane was used as the control in the olfactometric bioassays.

### 4.5. Chemotaxis Assays Using Olfactometers

**Figure 4:**
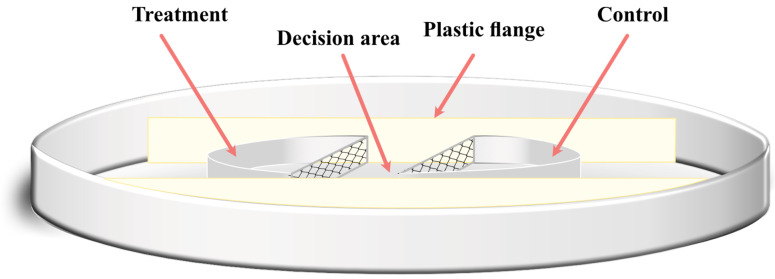
Schematic representation of the olfactometer designed to perform the behavioral assays with *Steinernema australe* and the selected compounds.

We designed an olfactometer (Figure 4) to evaluate the preference of *S. australe* for the selected compounds based on their chemotaxis. Our design comprised two plastic Petri dishes, with the external section consisting of the bottom piece of a 90 mm plastic Petri dish. The internal components included a 50 mm plastic Petri dish divided into halves. We set the halves in the center of the 90 mm plate opposed to each other, with a separation of 16 mm (decision area), flanged by two plastic pieces (80 mm × 10 mm) to fix the position. Both halves and the in-between portion were filled with ca. 2.5 g of moistened sand (15% humidity *w*/*w*). One half was randomly designated as the treatment area and the other half as the control. The section in between was considered the decision area. At five millimeters from the distal border of the treatment area, we added 50 µL of a standard solution of the selected compounds, and the control area received 50 µL of hexane. The olfactometers remained open to the air for 15 min to allow solvent evaporation. Then, 300 (±50) IJs of *S. australe* were suspended in one milliliter of distilled water and inoculated over the decision area. The labeled olfactometers were covered with a 90 mm Petri dish lid and randomly oriented in a dark room at 14 (±2 °C) for 15 h. The sand of each section (treatment and control) containing the IJs was individually recovered using 50 mL of tap water and kept in 500 mL plastic cups (Hefty^®^, 18 oz) until use. These cups were gently hand-shaken for 10 s and maintained for 45 min at 30° inclination for IJs decantation. Finally, the water containing the IJs was carefully recovered using a 1 mL plastic Pasteur pipette. A second wash of the sand was performed using 30 mL of tap water, repeating the previous procedure. The number of IJs collected was counted using a Nexius Zoom microscope (Euromex, The Netherlands). We performed 15 replicates for each compound and concentration. The olfactometer described in Figure 4 was considered a replicate.

### 4.6. Data Analysis

As a control for the chemotaxis trials, we conducted a complete set of bioassays using only hexane in the olfactometer and registered the IJs dispersion as a percentage of the total of individuals in each olfactometer area. Using authentic commercial standards of the volatiles as stimuli, we tested the IJs’ dispersion in the olfactometer. We determined the mean difference of the percentage for the treatment and control along with its bootstrap 95% confidence interval, by a two-sided permutation *t*-test with 5000 reallocations with 5000 resamples, through the DABEST package in R [71,72,73,74]. We plotted the data using GraphPad Prism version 8.4.3 (GraphPad Software, San Diego, CA, USA).

## 5. Conclusions

Based on the results of this study, we can state that blueberry plants cv. Legacy release volatile compounds in the soil through their roots. The nature of these compounds is modified due to the herbivory produced by larvae of the genus *Aegorhinus*. The dose–response curves showed that some of these compounds could elicit positive chemotaxis of *Steinernema australe*, an entomopathogenic nematode effective for controlling *Aegorhinus* larvae. These findings suggest that root volatiles could influence the performance of biological control agents for subterranean pests in blueberries.

## Figures and Tables

**Table 1:** Percentage of relative abundance (Mean ± SD) of chemicals identified in the volatile profile of *V. corymbosum* uninfested and infested roots with *A. superciliosus* larvae.

Compound	Relative Abundance in Roots (%)	
	Uninfested	Infested
*Terpenoids*		
4,8-Dimethyl-1,7-nonadien-4-ol	0.17 ± 0.12	0.08 ± 0.03
2-Carene	17.78 ± 7.03	24.94 ± 12.16
Limonene	3.60 ± 2.63	4.35 ± 1.45
Eucalyptol	4.02 ± 1.25	3.00 ± 1.40
Linalool	2.06 ± 1.04	2.98 ± 0.23
Myrcenol	4.91 ± 2.06	4.19 ± 1.1
cis-Myrtanol	1.63 ± 1.45	2.05 ± 1.61
α-Terpineol	29.21 ± 12.38	30.44 ± 8.57
*Esters*		
Isobutyl Isobutyrate	0.66 ± 0.35	0.63 ± 0.12
Methyl salicylate	17.89 ± 9.37	11.44 ± 5.53
Vinyl sorbate	0.86 ± 0.54	0.93 ± 0.11
*Aliphatic Hydrocarbons*		
1-nonyne	4.15 ± 2.63	2.53 ± 0.66
3-Ethenyl-1,4-pentadiene	0.06 ± 0.03	0.08 ± 0.03
*Alcohols*		
3-Butynol	0.24 ± 0.04	0.24 ± 0.05
10-undecyn-1-ol	5.21 ± 3.84	4.27 ± 3.54
*Ketones*		
3-Octanone	6.81 ± 6.16	7.11 ± 5.08
3-Hexanone	0.29 ± 0.04	0.28 ± 0.02
2-Hydroxy-2,4-dimethyl-3-pentanone	0.43 ± 0.23	0.44 ± 0.18

Volatile compounds were analyzed in triplicate, each sample comprised a mixture of the individual collection from six plants.

## Data Availability

Not applicable.
